# Changes in Food Consumption in Postmenopausal Women during the COVID-19 Pandemic: A Longitudinal Study

**DOI:** 10.3390/nu15153494

**Published:** 2023-08-07

**Authors:** Priscilla Rayanne E. Silva Noll, Monique G. Nascimento, Luiza Helena Costa Moreira Bayer, Juliana Zangirolami-Raimundo, José Antonio Orellana Turri, Matias Noll, Edmund Chada Baracat, José Maria Soares Junior, Isabel Cristina Esposito Sorpreso

**Affiliations:** 1 Department of Obstetrics and Gynecology, Faculdade de Medicina, Universidade de São Paulo, São Paulo 01246-903, Brazil; moniquegazoli@usp.br (M.G.N.); bayerluiza@gmail.com (L.H.C.M.B.); antonioturri@usp.br (J.A.O.T.); ecbaracat@gmail.com (E.C.B.); jose.msjunior@fm.usp.br (J.M.S.J.); icesorpreso@usp.br (I.C.E.S.); 2 Department of Education, Instituto Federal Goiano Campus Ceres, Ceres 76300-000, Brazil; matiasnoll@yahoo.com.br; 3 Faculdade de Saúde Pública, Universidade de São Paulo, São Paulo 01246-904, Brazil; 4 Faculdade de Medicina do ABC, Santo André 09060-650, Brazil; juliana.zangirolami@alumni.usp.br; 5 Department of Nutrition, Universidade Federal de Goiás, Goiânia 74605-080, Brazil

**Keywords:** coronavirus, food intake, eating habits, ultra-processed foods, industrialized food, food security, NOVA classification, menopausal symptoms

## Abstract

Studying the dietary habits and symptoms of postmenopausal women during situations such as the COVID-19 pandemic is important to mitigate long-term post-pandemic health problems. We compared the menopausal symptoms and food consumption in postmenopausal women before and during the COVID-19 pandemic. A longitudinal survey was conducted on postmenopausal Brazilian women between 2018 and 2021. The Kupperman–Blatt Menopausal Index, Women’s Health Questionnaire, and 24 h food recall were used. Of 274 women, 78 (28.5%) participated in the study during the COVID-19 pandemic. The intensity of the symptoms was lower during the pandemic than during the previous period (*p* < 0.05). Energy and processed food consumption were lower during the pandemic than before (*p* = 0.003 and *p* = 0.003, respectively). Milk and plain yogurt consumption were also lower (*p* = 0.043), while the consumption of sugar-sweetened beverages and sweet foods was higher (*p* = 0.007 and *p* = 0.001, respectively) during the pandemic. There was also a decrease in the consumption of proteins and lipids (*p* = 0.001 and *p* = 0.004, respectively). In conclusion, we found that postmenopausal women consumed sweet foods and sugar-sweetened beverages in higher quantities and had a lower consumption of milk and plain yogurt and processed foods during the pandemic than during the pre-pandemic period. Furthermore, decreases in energy and macronutrient consumption were observed.

## 1. Introduction

The novel coronavirus infection caused by SARS-CoV-2 was first reported in December 2019 in Wuhan, China, and spread globally [1]. Subsequently, the World Health Organization (WHO) declared Coronavirus disease (COVID-19) a global pandemic [2]. By February 2023, the viral infection had been confirmed in 758,390,564 people worldwide, causing 6,859,093 deaths [1]. The pandemic created enormous challenges in global and local health systems, the economy, and the food supply [3]. In light of these challenges, it is also crucial to recognize the importance of monitoring patients for reception, harm reduction, and maintenance of communication with health professionals, especially high-risk groups for mental health [4,5,6,7]. By prioritizing these measures, we can ensure the reception of appropriate care and support, thereby mitigating the potential long-term health consequences of the pandemic. Studies have reported worsening mental health issues such as depression, anxiety, and stress during the pandemic among different age groups [5,6,8]. Although the infection affects individuals across all age groups, older adults are particularly at high risk, as are individuals with medical conditions such as Non-Communicable Chronic Diseases (NCDs) [9,10]. This emphasizes the importance of monitoring postmenopausal women, especially in the presence of signs and symptoms related to this phase [7,8,11,12,13].

During the postmenopausal period, NCDs such as obesity, cardiovascular diseases, diabetes, and some cancers are diagnosed commonly [14,15]. Furthermore, several menopausal symptoms have been reported [16,17]. The most common symptoms include vasomotor symptoms, vaginal dryness, myalgia, sleep disturbances, sexual problems, urinary tract changes, mood changes, emotional instability, and dyspareunia [16,17]. The causes of these symptoms are multifactorial and include hormonal changes as well as cultural, psychological, and sociodemographic factors [16,17]. Quality of life and disease prevention are intrinsically related to a healthy environment during the postmenopausal period [18,19,20], including dietary patterns [18,19].

Studies have indicated that postmenopausal women tend to have a higher intake of sugars and refined carbohydrates during the menopausal transition [21]. Diets rich in highly processed (ultra-processed) foods, saturated fats, and sugars are reportedly associated with a greater intensity of psychological, vasomotor, urogenital, and somatic symptoms as well as sleep disturbances. In contrast, patterns involving the consumption of vegetables, whole grains, and unprocessed foods are associated with a lower intensity of menopausal symptoms [20,22].

The reduction in susceptibility to COVID-19 and its long-term consequences are influenced by access to and regular consumption of healthy foods [10]. However, studies have reported exacerbation of unhealthy dietary habits during the pandemic [3,23,24,25,26,27,28], particularly in middle-income countries and among individuals with lower education levels, indicating advanced dietary inequalities [24,25,26,27,29]. Understanding dietary habits and their relationship with postmenopausal symptoms during a pandemic is vital for healthcare planning. Research with this purpose can inform the healthcare system’s response to the needs of postmenopausal women after a global public health crisis. The healthcare system must accordingly reorient itself to meet the requirements of postmenopausal women to develop a sustainable and equitable long-term care system [8,10,12].

In line with the above concerns, this study compared menopausal symptoms and food consumption in postmenopausal women before and during the COVID-19 pandemic.

## 2. Materials and Methods

This longitudinal study was conducted between January 2018 and January 2021 at the Climacteric Gynecology Outpatient Clinic of the Faculdade de Medicina, Universidade de São Paulo.

### 2.1. Population and Sample

All the women who met the inclusion criteria at the outpatient clinic were invited to participate in this cohort study. The inclusion criteria were (a) postmenopausal diagnosis (12 consecutive months after the last menstrual period) [30] by the medical team of the Climacteric Clinic and (b) age ≥ 40 years. All participants were already being followed up by the medical team at the Climacteric Gynecology Outpatient Clinic for at least one year and already had an established postmenopausal diagnosis. The exclusion criteria were as follows: (a) surgical menopause; (b) current diagnosis of cancer and/or current use of chemotherapy; (c) untreated active parathyroid and thyroid endocrine disorders; (d) chronic renal failure; and (e) chronic liver failure.

A nonprobabilistic convenience sample was used in this study. The power and sample estimations were performed based on the international literature evaluating postmenopausal women’s total energy intake. Considering a power sample of 80%, an alpha error of 5%, and a magnitude of difference of 10%, the minimum number of patients to be included was 61 [20,31,32,33,34].

**Figure 1:**
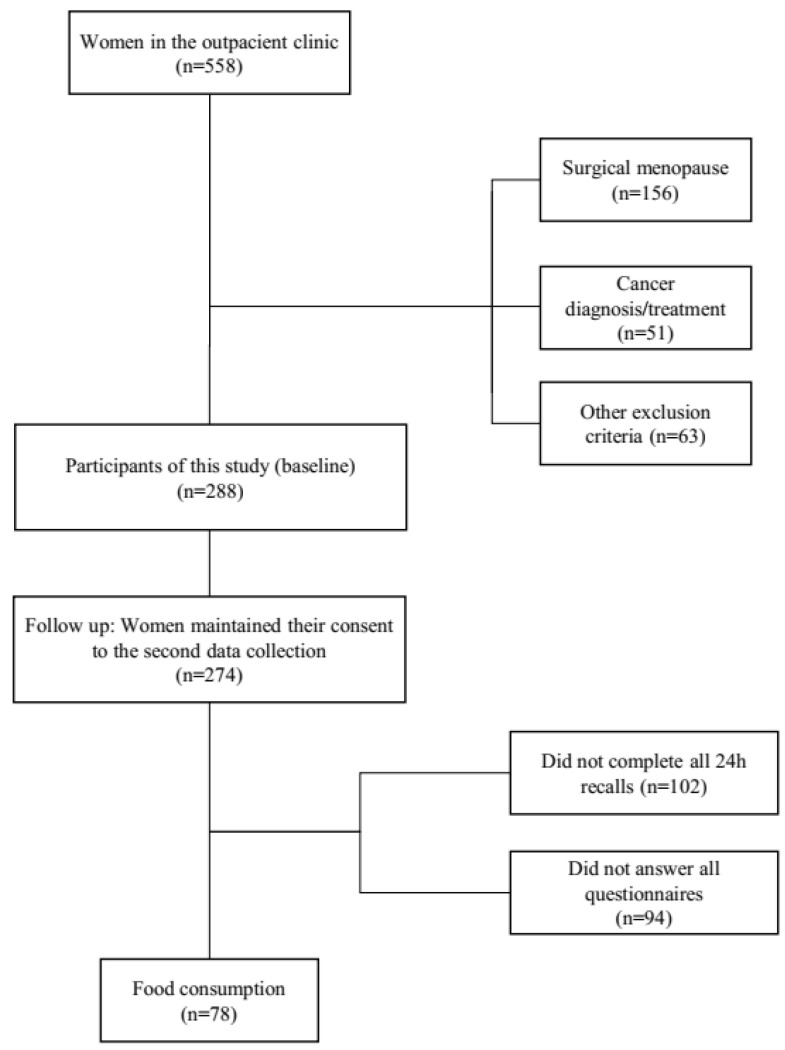
Flowchart of participants in the study cohort.

The first sample (baseline) comprised 288 postmenopausal women. In the second phase of data collection, 274 participants provided their consent to participate in this research. Of these, 78 participants chose to continue with the research and completed all the questionnaires during the second data collection (Figure 1). Noll et al. [20] presented more information regarding the characteristics of this cohort (baseline).

### 2.2. Ethical Aspects

All participants were informed of the study’s purpose, confidentiality of information, and data collection procedures. The participants received and accepted an approved verbal invitation to participate in the study. This study was approved by the FMUSP Research Ethics Committee (No. 2.427.142). Additionally, they signed two copies of an Informed Consent Form. This study was conducted in accordance with Resolution No. 466/2012 (Brazil) and the Declaration of Helsinki and was in compliance with the STROBE guidelines.

### 2.3. Data Collection and Measurement

The data were collected through individual interviews and questionnaires. The questionnaires included sociodemographic (age, ethnicity, marital status, monthly income, and education), clinical (age at menopause and menopause time), anthropometric, and lifestyle data (smoking and alcohol intake). Furthermore, the following questionnaires were administered: (1) International Physical Activity Questionnaire, (2) The Kupperman–Blatt Menopausal Index (K-BMI), (3) Women’s Health Questionnaire (WHQ), and (4) the 24 h dietary recall (24 h recalls). A trained and experienced team conducted the interviews and anthropometric measurements.

The anthropometric variables collected included height, body mass, and waist circumference (WC). Height was measured in centimeters using a stadiometer [35], and body mass was measured in kilograms using a calibrated digital electronic scale (Welmy brand, W110H 200 kg, Santa Bárbara d’Oeste, Brazil) [36,37]. Body Mass Index (BMI) was classified as underweight, normal weight, overweight, or obese [36]. WC was measured at the smallest curvature between the ribs and hip bone (iliac crest). If the smallest curvature was not visible, WC was measured at the midpoint between the iliac crest and the last rib [38].

Physical activity was assessed using the International Physical Activity Questionnaire (IPAQ) [39], a short version validated in Brazil [40]. The short version of the IPAQ assesses participants’ physical activity frequency (days/week) and duration (minutes/day) for walking, moderate exercise, and vigorous exercise in the past seven days. The physical activity levels were classified as the following: Sedentary: no physical activity for ≥10 continuous minutes in the last 7 days; Insufficiently active: engaged in physical activity for ≥10 continuous minutes in the last 7 days, but not enough time or frequency to be considered active; Active: met criteria for either vigorous physical activity on ≥3 days/week for ≥20 min/session, or moderate physical activity and/or walking on ≥5 days/week for ≥30 min, or accumulated at least 150 min of physical activity per week over ≥5 days. Subsequently, participants were grouped into two categories: non-active (sedentary and insufficiently active) and active [39].

The intensity of menopausal symptoms was evaluated using the K-BMI [41]. The K-BMI includes 11 symptom categories: vasomotor function, paresthesia, insomnia, nervousness, melancholy, vertigo, weakness, arthralgia/myalgia, headache, palpitations, and tingling. The total index score was calculated by adding the sum of all 11 categories, which was then classified as mild (<19 points), moderate (20–35 points), or severe (>35 points) [41].

The WHQ was used to assess the quality of life of women based on menopausal symptoms [42]. The WHQ comprises 36 questions divided into nine domains: depressive mood, somatic symptoms, anxiety/fear, vasomotor, sleep disorders, sexual behavior, menstrual symptoms, memory/concentration, and attractiveness. The menstrual symptom domain was excluded from the study because it evaluated postmenopausal women. Each question had a response scale from zero to four points. The points were added and divided by the number of questions corresponding to each domain, resulting in a binary scale from 0 to 1, where 0 represents a better quality of life or good health status, and 1 indicates a worse quality of life or poor health status [42].

Dietary intake was assessed based on three non-consecutive days of 24 h recalls [43,44,45] by trained dietitians supervised by a senior researcher. Food consumption data were collected on two weekdays and one weekend day [46]. The 24 h recall was adapted by Nupens/USP for food consumption data collection in a 24 h period (the previous day) [47]. The 24 h recalls collected information about foods and beverages ingested, quantity ingested, measures used at home, time and place of each meal, the origin of food preparation (food/ready preparation or homemade), and sugar added in each preparation [14]. The multiple-pass technique was used to reduce the recall bias [43,44].

According to the Reference Table for Measures of Foods Consumed in Brazil, the foods listed by the participants in the 24 h recalls were converted from household measures to grams (g) and milliliters (mL). The food amounts were then converted into kilocalories (kcal) using the Nutritional Composition Table of Foods Consumed in Brazil [48]. When participants reported having added sugar to their beverages (fruit juices, coffee, and tea), 10% of the entire volume of beverages consumed was considered added sugar [48,49].

Foods were classified into three groups according to the NOVA Classification, which is based on the degree and extent of industrial food processing: culinary preparations, processed foods, and ultra-processed foods [50]. Culinary preparations consist of recipes based on fresh or minimally processed foods, such as vegetables, fruits, meat, eggs, cereals, legumes, coffee, and milk. In the category of processed foods, there are items manufactured by the industry with the addition of common culinary ingredients such as salt, oil, fat, sugar, and vinegar. Examples include fresh bread, cheese, canned vegetables or meat, and cured meats. On the other hand, ultra-processed foods are industrial formulations derived from extracted substances found in food or synthesized in laboratories. These substances may include additives like flavor enhancers, carbonating agents, and emulsifiers. The ultra-processed category includes sugar-sweetened beverages, cookies, crackers, ice cream, sweets, ready-to-eat cakes, chocolate milk, fast food, sausages, and hamburgers [50].

The percentage contribution of calories from foods derived from culinary preparations, processed foods, and ultra-processed foods was calculated. Finally, the average intake of each nutrient and the percentage of consumption in each NOVA group were calculated based on the 3-day recall for comparison.

### 2.4. Follow-Up

A follow-up assessment was conducted between August 2020 and January 2021. Owing to the pandemic caused by the SARS-CoV-2 virus, quarantine was decreed in the state of São Paulo on 20 March 2020 [51]. Consequently, questionnaires for the second data collection round were administered remotely via Google Forms because in-person administration was not possible during this period. The remote questionnaire included questions about the diagnosis of COVID-19 and isolation, in addition to the K-BMI, WHQ, and IPAQ. The 24 h recalls were conducted over the phone during the same period. Of the total number of participating women, 28.5% (n = 78) agreed to continue participating in the study during the pandemic and completed questionnaires remotely.

At the time of reassessment, the population of Brazil had been living with the COVID-19 pandemic since March 2020, and vaccination protocols had not yet been implemented in the country [52,53]. The state of São Paulo had a death rate of 101.9 and 236.1 deaths per 100,000 inhabitants in the years 2020 and 2021, respectively, while the national rate was 93.14 and 201.5 deaths per 100,000 inhabitants. The region was engaged in social isolation, with the mandatory use of face masks; however, several basic services, such as healthcare and grocery stores, remained operational [52,53].

### 2.5. Data Analysis

Statistical analyses were performed using STATA 16 version SE software (StataCorp LLC, College Station, TX, USA). Distribution tests were conducted for socioeconomic, clinical, anthropometric, and lifestyle variables, as well as for questionnaire responses. Categorical and binary variables, as well as categorical variables created based on clinically relevant cutoff points for continuous quantitative variables, were analyzed using Chi-Square and Fisher’s exact tests to assess differences in the proportion of each category of the variable over the two periods between groups. For continuous variables, Wilcoxon tests were used as they are non-parametric. For all tests, *p*-values less than 0.05 were considered statistically significant [54].

## 3. Results

The socioeconomic, clinical, anthropometric, and lifestyle data for this cohort study are summarized in Table 1. Of the participants, 97.4% had not been diagnosed with COVID-19, and 79.5% were in isolation but working in person until January 2021. Of the 274 participants enrolled at the start of the pandemic, 78 (28.5%) continued to participate in the survey and responded to all the questionnaires using Google Forms. Therefore, the results of the statistical analysis are based on these participants (n = 78) (Table 2, Table 3 and Table 4).

There was a difference in the perception of menopausal symptoms before and during the pandemic, with the intensity of the total and stratified symptoms being lower during the pandemic (*p* < 0.05) (Table 2).

As shown in Table 3, energy and processed food consumption were lower during the pandemic than before the pandemic (*p* = 0.003 and *p* = 0.003, respectively). Regarding the healthy eating markers, the consumption of milk and plain yogurt decreased during the pandemic (*p* = 0.043). The consumption of sugar-sweetened beverages and sweet foods, which are unhealthy markers, was higher during the pandemic than before (*p* = 0.007 and *p* = 0.001, respectively). Calories from ultra-processed foods present in the women’s diet showed no significant difference before and during the pandemic.

Table 4 shows the participants’ nutrient consumption. The consumption of proteins and lipids was lower during the pandemic than during the previous period (*p* = 0.001, *p* = 0.004, respectively). The consumption of saturated and monounsaturated fatty acids also decreased (*p* = 0.011 and *p* = 0.006), in addition to the consumption of trans fatty acids (*p* < 0.002).

## 4. Discussion

The results of this study help better understand the health of postmenopausal women during the COVID-19 pandemic. To the best of our knowledge, this is the first study from Latin America to compare food consumption based on the degree of food processing, intensity of menopausal symptoms, and quality of life in this population, among other health aspects, before and during the pandemic period. Our main findings indicate higher consumption of sweet foods and sugar-sweetened beverages and lower consumption of milk, dairy products, and processed foods during the pandemic compared to the pre-pandemic period. Energy consumption, as well as protein and lipid macronutrients, decreased during the pandemic. In terms of menopausal symptoms, women reported lower total intensity and more specific symptoms, including vasomotor symptoms.

Our data indicate a greater consumption of sweet foods and sugar-sweetened beverages, both classified as ultra-processed foods, among women during the COVID-19 pandemic. The increase in the consumption of ultra-processed foods during the pandemic has been highlighted in other studies, particularly sweet foods consumed by women, compared to their usual intake [3,27,55,56,57,58,59]. Cohort studies conducted in France and the US have associated a greater desire for and intake of ultra-processed foods, especially those high in sugar, as potent distractors in participants with increased levels of stress [28,59]. In contrast, some studies have reported decreased consumption of packaged sweets, bakery products, and fast food during the COVID-19 lockdown [60]. Of note, the lockdown improved quality owing to increased time being allocated for cooking, better organization of household meals, and greater family support [61]. These findings are supported by those of other studies.

Inadequate eating habits have been associated with female sex, lower income, depressive symptoms, accumulation of responsibilities, and higher pre-lockdown consumption of ultra-processed foods in a French cohort [59]. It should be noted that the consumption of ultra-processed foods is evident in postmenopausal women, especially sweet foods and sugar-sweetened beverages, and their consumption is associated with a greater intensity of menopausal symptoms [20,22].

The WHO released dietary guidelines to be followed during the COVID-19 pandemic to promote healthy eating habits among individuals with the aim of strengthening the immune system and minimizing the consequences of COVID-19 [62]. In addition to avoiding sugar, fat, and salt, this recommendation focuses on the daily consumption of fresh and minimally processed foods, particularly fruits, vegetables, and legumes [62]. Our results indicate a reduction in the consumption of milk and natural yogurt during the pandemic as well as a decreasing trend in the consumption of legumes, although the difference was not significant. These findings corroborate data from studies conducted in several countries, such as Italy [3,60], France [59], the United States [28], and Brazil [55,56]. It is important to note that the consumption of these food items before the pandemic was still below the recommendations of the dietary guidelines, as was the consumption of fruits and vegetables [22]. An Italian study reported frequent consumption of cheese and dairy products (twice a day in 32.3% and 2–3 times a week in 44.7% of the participants) [3], which is distinct from our findings. Factors associated with lower consumption of fresh foods include limited access to more frequent purchases [3,59] and household food (in)security [63], making it difficult to purchase fresh foods. In developing countries, such as Latin American countries, existing social inequalities and those potentially aggravated by the pandemic further accentuate food (in)security [24,26,64].

Some studies have shown that spending more time cooking homemade meals [3,58,59,60,63] and eating together [63] may be associated with better eating habits. Cooking is indicative of healthy nutritional behavior, especially when cooking chores are shared among household members [3,61]. This activity involves several sociocultural aspects, including culinary skills, knowledge of local traditional foods, and commensality. Preparing meals at home is a habit in Latin America, particularly among women [65]. Although women in this study reported preparing meals at home, their consumption of healthy eating markers was insufficient. Furthermore, cooking habits may also have been a consequence of the absence of other options [59].

Our results indicate a lower caloric intake as well as lower consumption of proteins, carbohydrates, and lipids during the pandemic compared to the previous period. However, the diet quality did not seem to have improved, considering the high consumption of ultra-processed sweet foods and a lower intake of polyunsaturated fatty acids. Recent literature suggests that the intensification of existing social inequalities may be related to the intensification of household food (in)security, mainly in Latin American countries, such as Brazil [24,26,64,66].

According to another study conducted on Brazilian women, the intensity of menopausal symptoms was found to be lower during the pandemic than before [67], similar to the results of our study. These results may be related to the longer postmenopausal period of most participants, which classified them into the late phase. During this phase, there is a tendency towards a progressive and expected reduction in vasomotor symptoms; in contrast, in the late postmenopausal period there is a tendency towards an increase in the prevalence of genitourinary, sexual, cognitive, and cardiovascular diseases [16,68]. Furthermore, follow-up at a climacteric clinic may have contributed to the reduction in the intensity of symptoms. Other studies have pointed to the intensification of vasomotor and somatic symptoms, insomnia, and palpitations during the COVID-19 pandemic because of its psychological impact [7,69,70].

While the findings above provide novel insights, this study has some limitations that must be considered while interpreting our findings. The limitations of interviews and self-administered questionnaires include the possibility of underestimating or overestimating the reports, leading to social desirability and memory biases, which may introduce a validity bias. To overcome this limitation, the data collection team was adequately trained to avoid interference with self-reporting during the interviews. The women were assured of the anonymity and confidentiality of their responses. Additionally, a multiple-pass technique was used to conduct interviews. The online administration of self-reported questionnaires could also be another limitation of this study, as it may introduce biases due to self-selection and limited control over the survey environment. This format can also pose challenges for women with limited internet access, making it difficult for them to participate [64]. Nevertheless, we considered online surveys to be the most appropriate means to collect data due to pandemic restrictions to avoid participants’ exposure to the unnecessary risk of contagion from COVID-19. Additionally, the women who participated in this study were being followed up at the tertiary health service because of significant menopausal symptoms; therefore, the data cannot be generalized to the entire population. The number of study participants is also a key limitation to consider, as it can affect the association between the independent variables and the experienced period (before or during the pandemic). Regarding dietary habits, more 24 h recalls could have offered better estimates of regular diet intake [44]. However, three non-consecutive days is commonly used in the literature [43,44,45].

A key strength of this study was the assessment of food consumption by postmenopausal women specifically focusing on the degree of food processing, which is the current recommendation for the Brazilian population and in several other countries worldwide. It is important to evaluate food consumption based on the degree of food processing, owing to its impact on nutritional quality [71,72,73] and its relationship with NCDs [73,74,75], including cardiovascular diseases [71,72,73]. However, studies on this topic, particularly focusing on postmenopausal women, are scarce, especially in the context of the COVID-19 pandemic. Our results highlighted a lower intensity of menopausal symptoms during the pandemic period as well as changes in food consumption. These findings can contribute to improved healthcare planning for postmenopausal women to prevent and treat menopausal symptoms. The COVID-19 pandemic has underscored the need for health services to reorient themselves with the aim of minimizing the long-term consequences of CVDs and neoplasms in postmenopausal women [8,10,12,76].

## 5. Conclusions

Postmenopausal women had a higher consumption of sweet foods and sugar-sweetened beverages and a lower consumption of milk, dairy products, and processed foods during the pandemic than during the pre-pandemic period. There was also a decrease in energy and macronutrient intake; however, this did appear to contribute to the quality of the diet. The perceived intensity of menopausal symptoms, including vasomotor symptoms, was lower among postmenopausal women during the pandemic than during the pre-pandemic period.

## Figures and Tables

**Table 1:** Sociodemographic, clinical, lifestyle, and food consumption characteristics of postmenopausal women treated at the Climacteric Outpatient Clinic of the Gynecology Department, Hospital das Clínicas, Universidade de São Paulo, 2018–2021.

Sociodemographic and Clinical (n = 274)	Average (SD)/n (%)
Age	56.29 (6.22)
Age at menopause	48.13 (4.94)
Menopause time (years)	8.29 (6.62)
	n (%)
Ethnicity	
Nonwhite ^a^	128 (46.7)
White	146 (53.3)
Marital status	
Single/widow/divorced	129 (47.1)
Married or living with a partner	145 (52.9)
Individual monthly income ^b^	
≤2 minimum wages	175 (63.9)
>2 minimum wages	99 (36.1)
School education	
≤8 years	70 (26.8)
9 to 11 years	69 (26.3)
≥12 years	123 (46.9)
	Average (SD)
Anthropometric (n = 273)	
Body Mass Index (kg/m^2^)	28.40 (5.36)
Waist circumference (cm)	94.09 (12.99)
	n (%)
Body Mass Index	
Normal body weight	77 (28.2)
Overweight	105 (38.5)
Obesity	91 (33.3)
Life habits (n = 274)	
Physical activity	
Active	127 (46.4)
Inactive	147 (53.6)
Smoking	
No	227 (82.8)
Yes	47 (17.2)
Alcohol intake	
No	202 (73.7)
Yes	72 (26.3)
COVID-19 (n = 78) *	
Diagnosis of COVID-19 **	
No	76 (97.4)
Yes	2 (2.6)
Lockdown	
No	2 (2.6)
Yes, but still working in person	62 (79.5)
Yes	14 (17.9)

SD, standard deviation; ^a^ Black, yellow, and indigenous ethnic groups grouped; ^b^ Minimum salary BRL 998.00 (equivalent to 197 USD); * Women who participated in the collection before and during the COVID-19 pandemic; ** Until January 2021.

**Table 2:** Menopausal symptoms and quality of life of postmenopausal women treated at the Climacteric Outpatient Clinic of the Gynecology Department, Hospital das Clínicas, Universidade de São Paulo, 2018–2021, before and during the COVID-19 pandemic.

K-BMI	Ratings	Before Pandemic (n = 274)		During Pandemic (n = 78)		*p* *
		N	%	N	%	
Vasomotor	None	32	11.68	21	26.9	**<0.001**
	Mild	89	32.48	25	32.1	
	Moderate	58	21.17	22	28.2	
	Severe	95	34.67	10	12.8	
Paresthesia	None	80	29.2	47	60.26	**<0.001**
	Mild	116	42.34	19	24.36	
	Moderate	49	17.88	9	11.54	
	Severe	29	10.58	3	3.85	
Insomnia	None	41	14.96	23	29.49	**0.003**
	Mild	91	33.21	25	32.05	
	Moderate	43	15.69	16	20.51	
	Severe	99	36.13	14	17.95	
Nervousness	None	38	13.87	17	21.79	**0.034**
	Mild	104	37.96	31	39.74	
	Moderate	72	26.28	23	29.49	
	Severe	60	21.9	7	8.97	
Melancholy	None	41	14.96	27	34.62	**0.001**
	Mild	131	47.81	28	35.9	
	Moderate	62	22.63	19	24.36	
	Severe	40	14.6	4	5.13	
Vertigo	None	61	22.26	41	52.56	**<0.001**
	Mild	149	54.38	54	30.77	
	Moderate	35	12.77	11	14.1	
	Severe	29	10.58	2	2.56	
Weakness	None	59	21.53	34	43.59	**0.001**
	Mild	137	50	26	33.33	
	Moderate	51	18.61	14	17.95	
	Severe	27	9.85	4	5.13	
Arthralgia/Myalgia	None	30	10.95	18	23.08	0.05
	Mild	77	28.1	21	26.92	
	Moderate	62	22.63	17	21.79	
	Severe	105	38.32	22	28.21	
Headache	None	51	18.61	23	29.49	**<0.001**
	Mild	139	50.73	38	48.72	
	Moderate	48	17.52	17	21.79	
	Severe	36	13.14	0	0	
Palpitation	None	60	21.9	38	48.72	**<0.001**
	Mild	135	49.27	28	35.9	
	Moderate	55	20.07	11	14.1	
	Severe	24	8.76	1	1.28	
Tingling	None	48	21.9	33	42.31	**<0.001**
	Mild	144	49.27	35	32.05	
	Moderate	40	20.07	15	19.23	
	Severe	42	8.76	5	6.41	
Total	None	0	0	43	55.16	**<0.001**
	Mild	83	30.29	0	0	
	Moderate	141	51.46	30	38.46	
	Severe	50	18.25	5	6.41	
**WHQ**	**Before Pandemic (n = 78)**			**During Pandemic (n = 78)**		*p* **
	**Mean ± SD**		**Median (Q1–Q3)**	**Mean ± SD**	**Median (Q1–Q3)**	
Depressive mood	0.28 ± 0.26		0.14 (0–0.42)	0.28 ± 0.26	0.21 (0–0.42)	0.800
Somatic symptoms	0.51 ± 0.32		0.71 (0.14–0.71)	0.50 ± 0.28	0.42 (0.28–0.71)	0.195
Memory/concentration	0.57 ± 0.41		0.66 (0–1)	0.54 ± 0.37	0.66 (0.33–1)	0.512
Vasomotor	0.57 ± 0.53		1 (0–1)	0.57 ± 0.45	0.5 (0–1)	0.438
Anxiety/fear	0.39 ± 0.40		0.5 (0–0.75)	0.32 ± 0.35	0.25 (0–0.5)	0.094
Sexual behavior	0.61 ± 0.29		0.66 (0.33–1)	0.58 ± 0.30	0.66 (0.33–0.66)	0.707
Sleep disorders	0.62 ± 0.35		0.66 (0.33–1)	0.54 ± 0.39	0.66 (0.33–1)	0.369
Attractiveness	0.35 ± 0.47		0 (0–1)	0.41 ± 0.40	0.5 (0–0.5)	0.157

K-BMI: Kupperman–Blatt Menopausal Index; * Chi-Square Test; WHQ, Women’s Health Questionnaire; ** Wilcoxon test; Values in **bold** indicate significant data (*p* < 0.05).

**Table 3:** Energy consumption by degree of food processing of postmenopausal women treated at the Climacteric Outpatient Clinic of the Gynecology Department, Hospital das Clínicas, Universidade de São Paulo before and during the COVID-19 pandemic, 2018–2021.

Food Consumption	Before Pandemic (n = 78)		During Pandemic (n = 78)		*p* *
	Average ± SD	Median (Q1–Q3)	Average ± SD	Median (Q1–Q3)	
Total energy intake (kcal)	1726.04 ± 577.01	1802.81 (1371.08–2156.35)	1541.28 ± 604.07	1466.86 (1035.15–1918.58)	**0.003**
Degree of food processing (% of total daily energy intake)					
Culinary preparations	61.22 ± 14.45	60.78 (53.01–72.3)	63.14 ± 16.70	63.27 (51.81–74.60)	0.697
Processed foods	8.97± 6.44	8.75 (3.33–13.50)	5.88 ± 6.16	4.24 (0–9.71)	**0.003**
Ultra-processed foods	29.81 ± 14.42	29.38 (19.78–40.15)	31.18 ± 17.21	29.73 (18.29–41.77)	0.216
Healthy markers (g)					
Fruits	161.79 ± 109.42	153.5 (83.03–213.69)	135.22 ± 123.69	107.08 (44.96–180.01)	0.658
Vegetables	64.17 ± 78.93	38.54 (18.01–74.29)	55.70 ± 74.19	20.99 (9.05–83.53)	0.392
Legumes	78.93 ± 93.26	54.07 (0–109.75)	64.77 ± 78.09	38.82 (11.57–83.82)	0.063
Milk and plain yogurt	134.70 ± 124.02	116.10 (6.95–213.81)	106.67 ± 102.23	89 (2.99–168.9)	**0.043**
Unhealthy markers (g)					
Sugar-sweetened beverages	63.21 ± 81.68	29.93 (6.28–87.84)	66.99 ± 83.88	29.28 (0–89.8)	**0.007**
Sweet foods	184.15 ± 138.65	157.32 (62.51–273.34)	190.72 ± 227.02	128.12 (13.67–286.44)	**0.001**
Sausages	43.46 ± 75.71	0 (0–51.5)	26.65 ± 59.71	0 (0–19.94)	0.082
Fast food and ready meals	103.44 ± 189.54	15.17 (0–122.79)	101.64 ± 154.18	46.80 (0–131.61)	0.385

SD, standard deviation; * Wilcoxon test; values in **bold** indicate significant data (*p* < 0.05).

**Table 4:** Nutrient consumption among postmenopausal women treated at the Climacteric Outpatient Clinic of the Gynecology Department, Hospital das Clínicas, Universidade de São Paulo before and during the COVID-19 pandemic, 2018–2021.

	Before Pandemic (n = 78)		During Pandemic (n = 78)		*p* *
	Average ± SD	Median (Q1–Q3)	Average ± SD	Median (Q1–Q3)	
Total energy intake (kcal)	1726.04 ± 577.01	1802.81 (1371.08–2156.35)	1541.28 ± 604.07	1466.86 (1035.15–1918.58)	**0.003**
Nutrients (g)					
Proteins	64.89 ± 29.53	60.94 (45.30–85.87)	60.55 ± 28.19	54.19 (38.28–75.83)	**0.001**
Carbohydrates	212.92 ± 76.92	205.28 (159.43–266.13)	207.04 ± 79.52	195.67 (146.78–262.83)	0.079
Fibers	17.27 ± 7.14	15.74 (12.97–20.38)	16.76 ± 7.62	14.98 (12.25–20.32)	0.159
Lipids	56.39 ± 27.52	53.13 (35.00–73.88)	52.22 ± 25.34	47.79 (33.93–64.54)	**0.004**
Saturated Fatty Acids	21.82 ± 11.69	19.34 (13.75–28.05)	20.51 ± 10.97	17.52 (12.69–25.26)	**0.011**
Monounsaturated Fatty Acids	19.36 ± 9.87	16.33 (11.77–26.12)	17.76 ± 9.22	15.89 (11.00–21.89)	**0.006**
Polyunsaturated Fatty Acids	8.95 ± 4.68	8.59 (5.32–12.54)	8.33 ± 4.40	7.68 (4.80–14.16)	0.061
Trans Fatty Acids	2.27 ± 1.13	1.71 (1.26–3.06)	2.21 ± 1.34	1.75 (1.21–2.87)	**0.002**

SD, standard deviation; * Wilcoxon test; values in **bold** indicate significant data (*p* < 0.05).

## Data Availability

No linked research datasets were available for this study. The data will be made available upon request.
